# Optical–acoustic resonance fingerprinting of food-matrix perturbations in a phoxonic defect cavity

**DOI:** 10.1038/s41598-026-66770-0

**Published:** 2026-08-21

**Authors:** Arafa H. Aly

**Affiliations:** TH-PPM Group, Physics Department, Faculty of Sciences, Beni-Suef University, Beni Suef, 62111 Egypt

**Keywords:** Phoxonic crystal, Food-contamination sensing, Defect-mode resonance, Optical–acoustic fingerprinting, Food quality assessment, Transfer matrix method, Numerical modeling, Engineering, Optics and photonics, Physics

## Abstract

This study presents a compact defect-mode phoxonic crystal framework for the dual optical–acoustic characterization of contamination-induced changes in food media. The proposed sensing platform consists of a food-sensitive defect cavity positioned between two Bragg mirrors, where perturbations in the effective optical and acoustic properties of the defect medium generate complementary resonance responses. The optical response was evaluated using the transfer matrix method, whereas the acoustic response was described through an effective defect-mode resonance model. Contamination was represented by a normalized perturbation parameter that modifies the refractive index, optical loss, density, and sound velocity of the investigated food media. Increasing contamination produced a systematic optical resonance redshift and acoustic resonance downshift, confirming that the two channels probe distinct but related physical properties. The optical sensitivity varied from 4.4025 to 7.6953 nm/fraction, while the acoustic sensitivity ranged from 77.901 to 105.314 kHz/fraction, with highly linear behavior throughout the studied range. Under the assumed readout noise floors, the estimated limits of detection were 0.00390–0.03716 fraction for the optical channel and 0.01558–0.01991 fraction for the acoustic channel. Optical–acoustic fingerprinting and a relative phoxonic contamination index were further introduced to compare the dual-channel responses of different food samples. The combined response increased the mean normalized endpoint separation to 0.761, representing a 46.7% improvement over the best single-channel result. Additional analyses of Q-factor degradation, representative field localization, thickness tolerance, surface roughness, phase response, and group delay were included to clarify the sensing mechanism and evaluate structural robustness. The results indicate that defect-mode phoxonic crystals can provide a useful numerical platform for contamination-sensitive food characterization. Nevertheless, the present work remains an effective numerical proof-of-concept, and experimental implementation, calibration, and validation using real contaminated food samples are required before direct biochemical or microbiological detection can be established.

## Introduction

Food contamination poses a persistent challenge to public health, food-quality control, and rapid screening. It can result from microbial activity, chemical residues, adulteration, processing-related defects, or physicochemical changes during storage and transportation. Although conventional analytical techniques can provide accurate identification, they commonly depend on laboratory infrastructure, skilled personnel, sample preparation, and extended analysis times. These limitations continue to motivate the development of compact, rapid, label-free, and physically interpretable sensing approaches capable of detecting subtle changes in food media before substantial quality or safety deterioration occurs.

Photonic crystals are periodic dielectric structures designed to control and localize light through the formation of photonic bandgaps. The concepts of inhibited spontaneous emission in periodic media^1^ and photon localization in disordered structures^2^ established the foundation for manipulating light–matter interactions in engineered optical systems. In photonic crystal sensors, a localized defect mode within the bandgap acts as a sensitive spectral feature. Changes in refractive index, absorption, or optical path length within the sensing region modify the defect-mode condition and produce measurable resonance shifts, enabling label-free chemical and biological sensing.

Phoxonic crystals broaden this sensing concept by integrating optical and acoustic responses within a single framework. Previous studies have demonstrated efficient control and manipulation of acoustic-wave propagation in phononic crystal structures^3^, structures with simultaneous photonic and phononic bandgaps^4^, slow-photon and slow-acoustic-phonon waveguides^5^, optomechanical cavities^6^, phoxonic cavities with photon–phonon interaction^7^, and related cavity-optomechanics platforms^8^. These developments support the use of phoxonic structures for dual-channel sensing. In food-related applications, the two channels provide complementary information because the optical response is mainly influenced by refractive-index and optical-loss variations, whereas the acoustic response depends on density, compressibility, and sound velocity. Accordingly, contamination-induced changes can be characterized through two distinct physical signatures rather than by an optical response alone. Investigations of three-dimensional phoxonic structures^9^ and guided optical–acoustic waves in phoxonic slabs^10^ further demonstrate the potential of these systems for compact multiparametric sensing.

Several photonic crystal platforms have already been explored for food-related detection. A defect-engineered one-dimensional photonic crystal was proposed for high-resolution urine sensing through refractive-index-dependent spectral shifts^11^. A hybrid plasmonic–photonic crystal was experimentally demonstrated for optical detection of bacterial contaminants^12^, while ternary photonic crystal structures were investigated for enhanced milk-fat sensing^13^. Recent reviews have also highlighted the growing importance of photonic crystal materials in food detection and chemical-hazard analysis^14,15^. These studies confirm the suitability of periodic optical media for food-quality and food-safety applications.

Despite this progress, most reported photonic crystal food sensors depend mainly on a single optical readout, such as resonance wavelength shift, color change, or intensity variation. Although sensitive, single-channel responses can be affected by matrix complexity, turbidity, optical loss, temperature variation, or fabrication imperfections. Broader biosensing studies have also emphasized the need for rapid, portable, and robust platforms, including optical, electrochemical, microfluidic, and nanomaterial-based approaches, to improve the reliability of food-contaminant detection^16–24^. In this context, a dual optical–acoustic phoxonic strategy may offer a more informative sensing route by combining optical resonance redshift with acoustic resonance downshift.

High-Q resonances, defect-mode localization, and refractive-index tracking are central to photonic crystal and optical resonator biosensing. Photonic crystal sensors have been widely reviewed as compact and sensitive optical platforms^25^, and point-of-care biosensing studies have shown their ability to support label-free readout and strong light–matter interaction^26^. Surface photonic crystal biosensors and two-dimensional photonic crystal biosensors have further demonstrated the value of resonant structures for biological and biochemical detection^27,28^. Moreover, high-sensitivity photonic crystal cavities and one-dimensional defect-layer sensors have shown that localized modes can respond strongly to small changes in the defect or analyte region^29,30^.

Based on these developments, this study proposes a compact one-dimensional defect-based phoxonic crystal sensor for dual-channel monitoring of food-contamination-induced property changes. The structure consists of two Bragg mirrors surrounding a food-sensitive defect cavity. Contamination is modeled through normalized perturbations in the optical and acoustic properties of the defect layer, including refractive index, optical loss, density, and sound velocity. The optical response is calculated using the transfer matrix method, while the acoustic response is described using an effective defect-mode resonance model. The sensor performance is evaluated through optical resonance redshift, acoustic frequency downshift, Q-factor degradation, sensitivity, limit of detection, field confinement, thickness tolerance, surface roughness, phase response, and group delay.

The main contribution of this work is the integration of optical and acoustic defect-mode responses within one compact phoxonic framework for food-contamination-sensitive measurement and characterization. Unlike conventional single-readout photonic sensors, the proposed platform simultaneously evaluates optical redshift, acoustic downshift, resonance-quality degradation, optical–acoustic fingerprinting, and robustness indicators. The present study provides an effective numerical proof-of-concept and a physically interpretable framework for developing compact dual-channel phoxonic sensors for food-quality monitoring and contamination characterization. However, experimental calibration, implementation, and validation using real contaminated food samples are required before direct biochemical or microbiological identification can be claimed; these aspects are beyond the scope of the present work and will be addressed in future studies. In the proposed configuration, the investigated liquid food is introduced into the central defect cavity and acts as the optical and acoustic sensing medium.

## Theory and numerical simulation

This section summarizes the governing equations used in the compact numerical model. The optical channel is calculated using the transfer matrix method (TMM), while the acoustic channel is treated using an effective defect-mode resonance approximation. Food contamination is represented by a normalized concentration parameter, (C), and the model should be interpreted as an effective numerical framework rather than a direct biochemical or microbiological identification method.

### Defect-mode phoxonic configuration

The one-dimensional defect-based phoxonic crystal is written as.

1 $${\left(AB\right)}^{N}D{\left(BA\right)}^{N}$$

Where *A* and *B* are the low- and high-index Bragg layers, respectively, *D* is the food-sensitive defect cavity, and *N* is the number of periods on each side of the defect. The Bragg layers are selected using quarter-wave optical thicknesses,

2 $${d}_{A}=\frac{{\lambda}_{0}}{4{n}_{A}}$$

3 $${d}_{B}=\frac{{\lambda}_{0}}{4{n}_{B}}$$

where ${d}_{A}$ and ${d}_{B}$ are the layer thicknesses, ${\lambda}_{0}$ is the design wavelength, and ${n}_{A}$ and ${n}_{B}$ are the refractive indices of layers *A* and *B*. The defect layer is chosen as a half-wave cavity,

4 $${d}_{D}=\frac{{\lambda}_{0}}{2{n}_{D}^{0}}$$

where ${d}_{D}$ is the defect thickness and ${n}_{D}^{0}$ is the reference effective refractive index of the defect cavity.

### Optical transfer-matrix model

For each optical layer *j*, the characteristic matrix is

5 $${\mathbf{M}}_{j}=\left[\begin{array}{cc}\mathrm{c}\mathrm{o}\mathrm{s}{\delta}_{j}& i\mathrm{s}\mathrm{i}\mathrm{n}{\delta}_{j}/{q}_{j}\\ i{q}_{j}\mathrm{s}\mathrm{i}\mathrm{n}{\delta}_{j}& \mathrm{c}\mathrm{o}\mathrm{s}{\delta}_{j}\end{array}\right]$$

where the phase thickness is

6 $${\delta}_{j}=\frac{2\pi {n}_{j}{d}_{j}}{\lambda }$$

At normal incidence, the optical admittance is

7 $${q}_{j}={n}_{j}$$

The total transfer matrix of the multilayer stack is

8 $$\mathbf{M}=\prod_{j=1}^{m}{\mathbf{M}}_{j}=\left[\begin{array}{cc}{M}_{11}& {M}_{12}\\ {M}_{21}& {M}_{22}\end{array}\right]$$

The complex transmission coefficient is

9 $$t=\frac{2{n}_{i}}{{n}_{i}{M}_{11}+{n}_{i}{n}_{e}{M}_{12}+{M}_{21}+{n}_{e}{M}_{22}}$$

and the optical transmittance is

10 $${T}_{o}=\mathrm{R}\mathrm{e}\left(\frac{{n}_{e}}{{n}_{i}}\right){\left|t\right|}^{2}$$

where ${n}_{i}$ and ${n}_{e}$ are the refractive indices of the incident and exit media, respectively.

### Effective optical contamination model

The normalized contamination level is defined as

11 $$0\le C\le 1$$

The contamination-dependent refractive index and extinction coefficient of the defect layer are modeled as

12 $${n}_{D}\left(C\right)={n}_{D}^{0}+{\alpha}_{n}C$$

13 $${k}_{D}\left(C\right)={k}_{D}^{0}+{\alpha}_{k}C$$

The complex defect refractive index is therefore

14 $${\widetilde{n}}_{D}\left(C\right)={n}_{D}\left(C\right)-i{k}_{D}\left(C\right)$$

where ${n}_{D}^{0}$ and ${k}_{D}^{0}$ are the clean-state refractive index and extinction coefficient, while ${\alpha}_{n}$ and ${\alpha}_{k}$ are effective optical perturbation coefficients. Increasing ${n}_{D}\left(C\right)$ increases the defect optical path length and produces a redshift, whereas increasing ${k}_{D}\left(C\right)$ introduces additional optical loss.

### Optical resonance, quality factor, sensitivity and detection limit

The optical resonance wavelength is extracted at the maximum transmission peak,

15 $${\lambda}_{r}=\lambda {|}_{{T}_{o}={T}_{o,m}}$$

and the contamination-induced wavelength shift is

16 $$\Delta \lambda \left(C\right)={\lambda}_{r}\left(C\right)-{\lambda}_{r}\left(0\right)$$

The optical quality factor is

17 $${Q}_{o}=\frac{{\lambda}_{r}}{\Delta {\lambda}_{1/2}}$$

where $\Delta {\lambda}_{1/2}$ is the optical full width at half maximum. The optical sensitivity is

18 $${S}_{o}=\frac{d{\lambda}_{r}}{dC}$$

For discrete simulated data, the resonance wavelength is fitted as

19 $${\lambda}_{r}\left(C\right)={S}_{o}C+{b}_{o}$$

The total optical uncertainty is

20 $${\sigma}_{o}=\sqrt{{\sigma}_{fo}^{2}+{\sigma}_{io}^{2}}$$

and the optical limit of detection is

21 $${L}_{o}=\frac{3{\sigma}_{o}}{\left|{S}_{o}\right|}$$

where ${\sigma}_{fo}$ is the fitting residual uncertainty, ${\sigma}_{io}$ is the instrumental noise floor, and ${L}_{o}$ denotes the optical detection limit.

### Effective acoustic defect-mode model

The contamination-dependent density and sound velocity of the defect layer are represented as

22 $${\rho}_{D}\left(C\right)={\rho}_{D}^{0}+{\alpha}_{\rho }C$$

23 $${v}_{D}\left(C\right)={v}_{D}^{0}-{\alpha}_{v}C$$

where ${\rho}_{D}^{0}$ and ${v}_{D}^{0}$ are the clean-state density and sound velocity of the defect medium, and ${\alpha}_{\rho }$ and ${\alpha}_{v}$ are effective acoustic perturbation coefficients. The acoustic defect-mode resonance frequency is modeled as

24 $${f}_{a}\left(C\right)={f}_{0}\left(\frac{{v}_{D}\left(C\right)}{{v}_{D}^{0}}\right)\left[\frac{1}{\sqrt{{\rho}_{D}\left(C\right)/{\rho}_{D}^{0}}}\right]$$

This relation reflects the physical trend that increasing density and decreasing sound velocity shift the acoustic resonance toward lower frequencies.

The acoustic transmission is described by a Lorentzian-type defect-mode response,

25 $${T}_{a}\left(f,C\right)=\frac{{A}_{a}\left(C\right)}{1+4{\left[\frac{f-{f}_{a}\left(C\right)}{{\Gamma}_{a}\left(C\right)}\right]}^{2}}+{T}_{b}$$

with the effective contamination-dependent linewidth

26 $${\Gamma}_{a}\left(C\right)={\Gamma}_{a}^{0}\left(1+\beta C\right)$$

where ${T}_{a}\left(f,C\right)$ is the acoustic transmission, ${A}_{a}\left(C\right)$ is the acoustic resonance amplitude, ${f}_{a}\left(C\right)$ is the acoustic defect-mode resonance frequency, ${\Gamma}_{a}\left(C\right)$ is the acoustic linewidth at contamination level *C*, ${\Gamma}_{a}^{0}$ is the clean-state acoustic linewidth, *β* is the effective linewidth-broadening coefficient, and ${T}_{b}$ is a small acoustic background term.

In the present work, the term “phoxonic” refers to an effective dual optical–acoustic defect-mode sensing framework, while a comprehensive phononic-crystal analysis, including phononic band structures, acoustic-field distributions, and elastic-mode calculations, is beyond the scope of this numerical study and will be addressed in future work.

### Acoustic resonance, quality factor, sensitivity and detection limit

The acoustic resonance frequency is extracted as

27 $${f}_{r}=f{|}_{{T}_{a}={T}_{a,m}}$$

The acoustic frequency shift is

28 $$\Delta f\left(C\right)={f}_{r}\left(C\right)-{f}_{r}\left(0\right)$$

The acoustic quality factor is

29 $${Q}_{a}=\frac{{f}_{r}}{\Delta {f}_{1/2}}$$

where $\Delta {f}_{1/2}$ is the acoustic full width at half maximum. The acoustic sensitivity is

30 $${S}_{a}=\frac{d{f}_{r}}{dC}$$

For discrete simulated data, the resonance frequency is fitted as

31 $${f}_{r}\left(C\right)={S}_{a}C+{b}_{a}$$

Because the acoustic resonance downshifts with contamination, ${S}_{a}$ is generally negative; therefore, $\left|{S}_{a}\right|$ is used for sensitivity comparison. The total acoustic uncertainty is

32 $${\sigma}_{a}=\sqrt{{\sigma}_{fa}^{2}+{\sigma}_{ia}^{2}}$$

and the acoustic detection limit is

33 $${L}_{a}=\frac{3{\sigma}_{a}}{\left|{S}_{a}\right|}$$

where ${\sigma}_{fa}$ is the acoustic fitting residual uncertainty, ${\sigma}_{ia}$ is the acoustic instrumental noise floor, and ${L}_{a}$ denotes the acoustic detection limit.

### Linearity and dual-channel decision score

The coefficient of determination is calculated as

34 $${R}^{2}=1-\frac{\sum_{i}{\left({y}_{i}-{\widehat{y}}_{i}\right)}^{2}}{\sum_{i}{\left({y}_{i}-{y}_{m}\right)}^{2}}$$

where ${y}_{i}$ are the simulated values, ${\widehat{y}}_{i}$ are the fitted values, and ${y}_{m}$ is the mean value. The normalized optical and acoustic detection scores are

35 $${\eta}_{o}\left(C\right)=\frac{\left|\Delta \lambda \left(C\right)\right|}{\Delta {\lambda}_{T}}$$

36 $${\eta}_{a}\left(C\right)=\frac{\left|\Delta f\left(C\right)\right|}{\Delta {f}_{T}}$$

The final dual-channel score is

37 $${\eta}_{d}\left(C\right)=\mathrm{m}\mathrm{a}\mathrm{x}\left[{\eta}_{o}\left(C\right),{\eta}_{a}\left(C\right)\right]$$

A sample is classified as contaminated if either channel exceeds its threshold,

38 $$\left|\Delta \lambda \left(C\right)\right|\ge \Delta {\lambda}_{T} \mathrm{o}\mathrm{r} \left|\Delta f\left(C\right)\right|\ge \Delta {f}_{T}$$

### Representative electric-field confinement

The normalized electric-field intensity is

39 $${\left|E\left(z\right)\right|}_{n}^{2}=\frac{{\left|E\left(z\right)\right|}^{2}}{\mathrm{m}\mathrm{a}\mathrm{x}\{{\left|E\left(z\right)\right|}^{2}\}}$$

The longitudinal localization envelope is represented as

40 $${E}_{e}\left(z\right)=\mathrm{e}\mathrm{x}\mathrm{p}\left[-\gamma \frac{\left|z-{z}_{D}\right|}{L}\right]$$

The standing-wave modulation is

41 $${E}_{s}\left(z\right)={a}_{E}+{b}_{E}{\mathrm{c}\mathrm{o}\mathrm{s}}^{2}\left(\frac{2\pi {n}_{j}{z}_{l}}{{\lambda}_{r}}\right)$$

The representative two-dimensional field map is

42 $${\left|E\left(z,x\right)\right|}^{2}={\left|E\left(z\right)\right|}^{2}\mathrm{e}\mathrm{x}\mathrm{p}\left[-{\left(\frac{x}{w}\right)}^{2}\right]$$

where ${z}_{D}$ is the defect-center position, *L* is the total structure thickness, ${z}_{l}$ is the local position inside a layer, *w* is the effective transverse width, and ${a}_{E}$ and ${b}_{E}$ are standing-wave weighting constants. The field profiles are representative localization maps and are not full-wave FEM or FDTD simulations.

### Thickness tolerance and surface roughness

For Monte Carlo thickness-tolerance analysis, the perturbed layer thickness is

43 $${d}_{j}^{*}={d}_{j}\left[1+\frac{\tau }{100}\left(2r-1\right)\right]$$

where *τ* is the thickness-tolerance percentage and *r* is a uniformly distributed random number between 0 and 1. The tolerance-induced resonance uncertainty is

44 $${\sigma}_{\lambda }\left(\tau \right)=\mathrm{s}\mathrm{t}\mathrm{d}\left({\lambda}_{r}^{\left(1\right)},{\lambda}_{r}^{\left(2\right)},\dots ,{\lambda}_{r}^{\left({N}_{M}\right)}\right)$$

The normalized peak transmission under surface roughness is

45 $${T}_{p}\left(\sigma \right)=\mathrm{e}\mathrm{x}\mathrm{p}\left(-{a}_{R}{\sigma }^{2}\right)$$

The normalized Q-factor is

46 $${Q}_{R}\left(\sigma \right)=\frac{1}{1+{b}_{R}\sigma +{c}_{R}{\sigma }^{2}}$$

and the normalized linewidth broadening is

47 $${W}_{R}\left(\sigma \right)=\frac{1}{{Q}_{R}\left(\sigma \right)}$$

where *σ* is the root-mean-square surface roughness, and ${a}_{R}$, ${b}_{R}$, and ${c}_{R}$ are effective roughness-degradation coefficients. This model captures the expected roughness-induced scattering trend: reduced peak transmission, reduced Q-factor, and increased linewidth.

#### Phase response and group delay

The transmission phase is calculated from the complex transmission coefficient after phase unwrapping as

48 $$\phi \left(\lambda \right)=\mathcal{U}\{\mathrm{a}\mathrm{r}\mathrm{g}\left[t\left(\lambda \right)\right]\}$$

The angular frequency is

49 $$\omega =\frac{2\pi c}{\lambda }$$

The group delay is

50 $${\tau}_{g}=-\frac{d\phi }{d\omega }$$

A local group-delay maximum near the defect resonance indicates enhanced optical localization and a longer effective light residence time inside the defect region.

## Results and discussion

### Physical interpretation of the dual-channel sensing concept

The proposed model converts contamination-induced changes in the food-sensitive defect cavity into two physically different readouts. The optical branch responds mainly to changes in the effective refractive index and optical loss, whereas the acoustic branch responds to changes in effective density and sound velocity. This separation is important because the two channels do not measure the same property. The optical signal is dominated by changes in the cavity optical path length, while the acoustic signal is governed by changes in mechanical inertia and wave speed. Therefore, the same contamination process produces two complementary signatures rather than a duplicated response.

The central defect layer plays a key role in the sensing mechanism. The periodic Bragg mirrors confine the defect mode, and the food sample perturbs the localized cavity region. As defined in the Mathematical Formulation section, the contamination parameter modifies the optical and acoustic properties of the defect layer. In the results below, the optical resonance shifts to longer wavelength, whereas the acoustic resonance shifts to lower frequency. These opposite trends are physically consistent with the assumed increase in optical path length and the simultaneous reduction of acoustic resonance frequency.

**Fig. 1:**
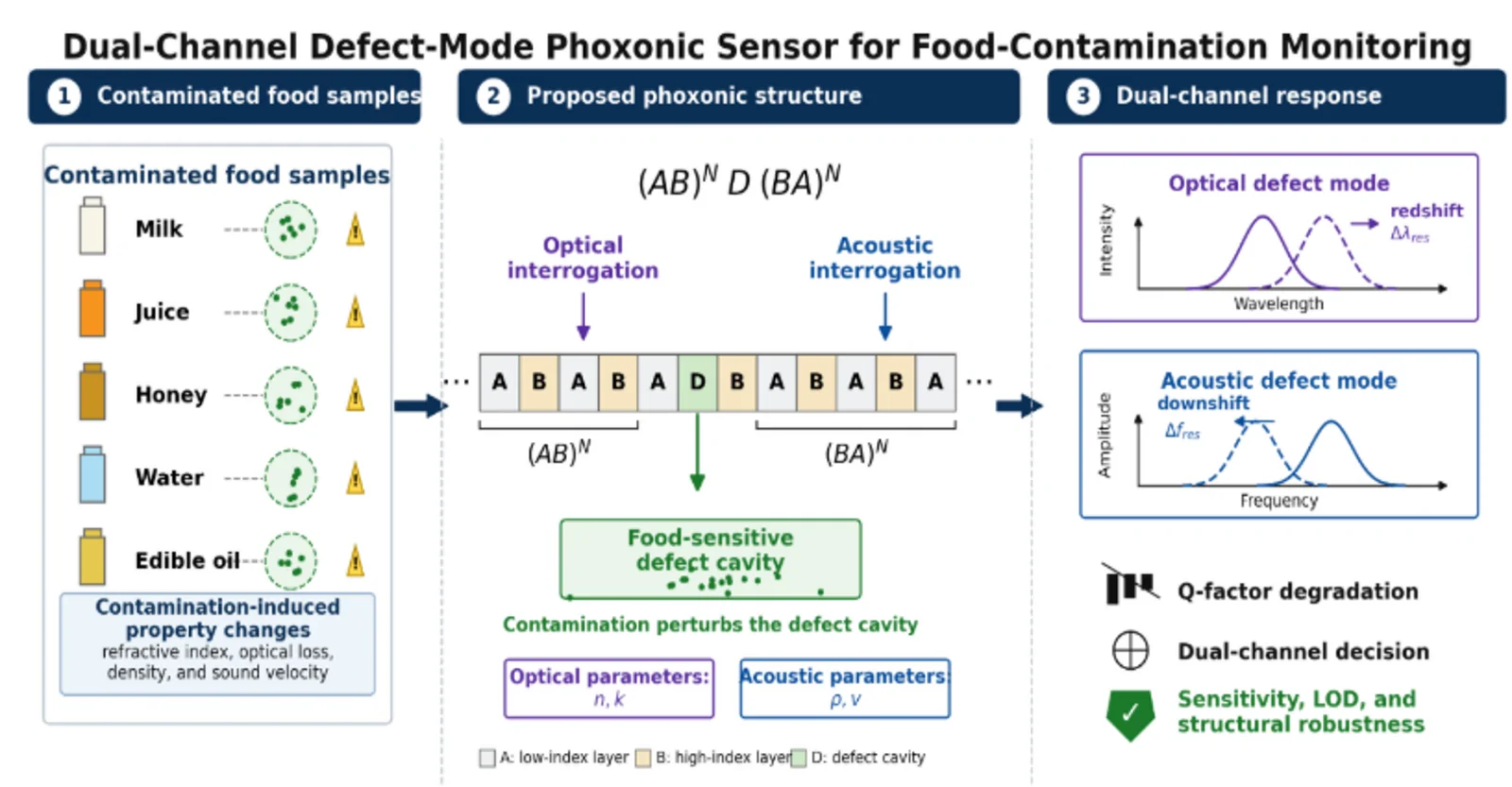
Schematic illustration of the proposed defect-mode phoxonic food-contamination sensor, showing how contamination-induced changes in the defect cavity modify both optical and acoustic properties, producing optical resonance redshift and acoustic frequency downshift for dual-channel detection.

The schematic representation of the proposed dual-channel defect-mode phoxonic sensor for food-contamination detection is shown in Fig. 1.

The scheme summarizes the sensing concept in three connected stages. First, different food media, including milk, juice, honey, water, and edible oil, may undergo contamination-induced physicochemical changes such as variations in refractive index, optical loss, density, and sound velocity. Second, these changes are introduced into the food-sensitive defect cavity D located between two periodic Bragg mirrors in the form (AB)^N^ D (BA)^N.^. The defect layer acts as the active sensing region, where both optical and acoustic waves interact strongly with the perturbed food medium. Third, the contamination-induced perturbation produces two complementary resonance signatures: an optical redshift caused mainly by refractive-index/loss variation, and an acoustic downshift caused mainly by density increase and sound-velocity reduction. In addition to resonance displacement, Q-factor degradation, dual-channel decision, sensitivity, Limit of Detection (LOD), and robustness analyses are used to provide a more complete interpretation of the sensing response.

### Optical defect-mode spectra and redshift response

**Fig. 2:**
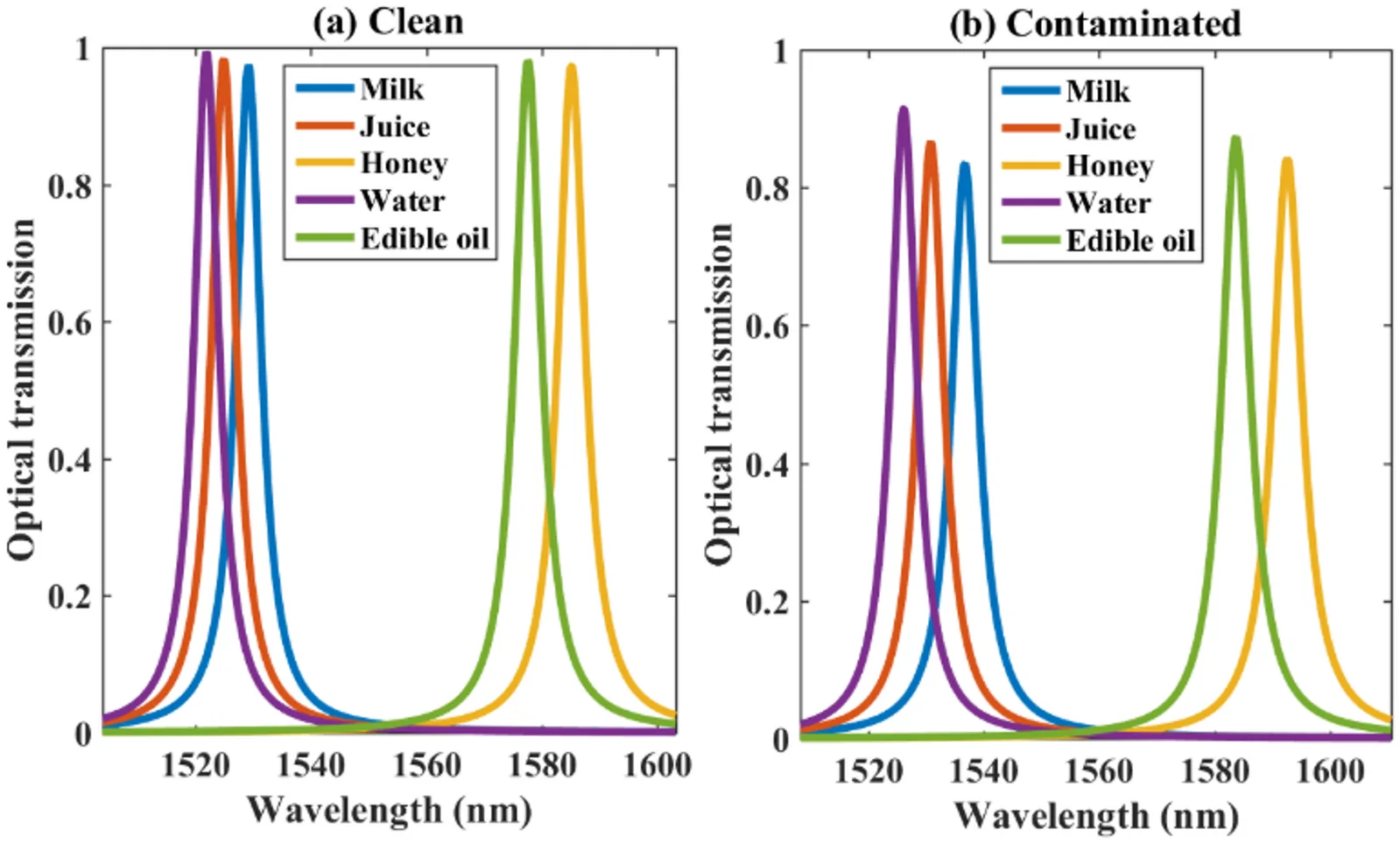
Optical transmission spectra of the proposed defect-mode phoxonic sensor for clean and contaminated food samples. The contaminated spectra exhibit resonance redshift and reduced peak sharpness due to contamination-induced changes in the effective refractive index and optical loss of the defect cavity.

Figure 2 compares the optical transmission spectra of the clean and contaminated food samples. The narrow peaks correspond to localized defect modes formed within the photonic stopband by the central cavity. As the contamination level increases, the resonances shift toward longer wavelengths because the increase in the effective refractive index of the defect layer enlarges its optical path length and changes the resonance phase condition. The contaminated spectra also exhibit broader and less sharp peaks, reflecting the additional optical loss introduced through the extinction-coefficient perturbation. Thus, the optical response is characterized by both the resonance redshift and the associated reduction in resonance quality.

### Acoustic defect-mode spectra and downshift response

**Fig. 3:**
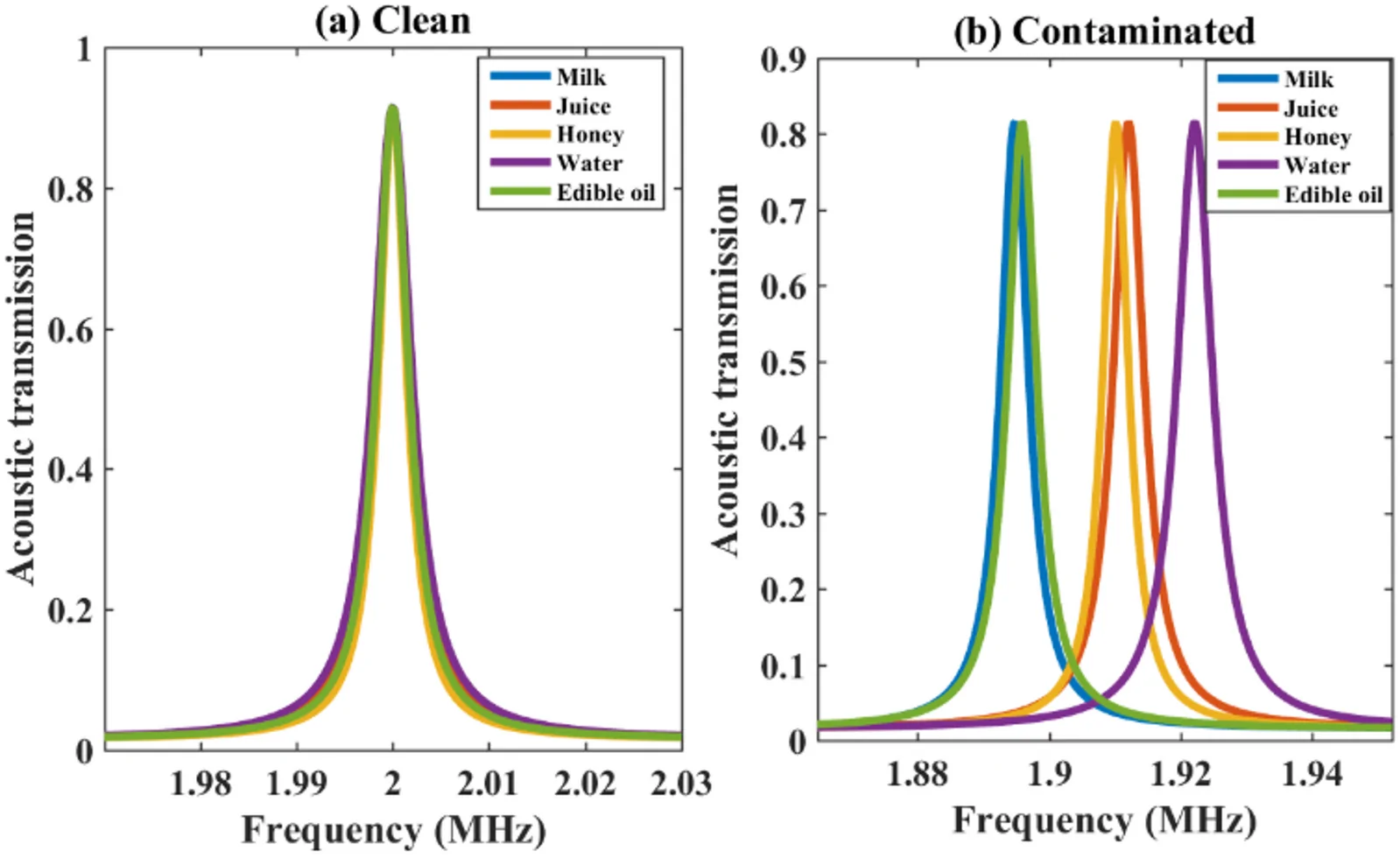
Acoustic transmission spectra for clean and contaminated food samples. The contaminated spectra show acoustic resonance downshift, reflecting the combined effect of increased effective density and reduced sound velocity in the defect region.

Figure 3 presents the acoustic defect-mode spectra for the clean and contaminated states. In the clean condition, the resonance peaks remain near the reference acoustic frequency, providing a consistent baseline for comparison among the investigated food media. With increasing contamination, the resonances shift systematically toward lower frequencies. This downshift results from the combined effects of an increase in effective density and a decrease in sound velocity, which increase the acoustic inertia and reduce the wave-propagation speed within the defect medium, respectively. Consequently, the acoustic channel probes mechanical-property variations that are distinct from the optical response. It therefore serves as a complementary readout that can help confirm contamination-sensitive changes, particularly when the optical signal may be affected by turbidity, absorption, or scattering.

### Resonance tracking, sensitivity, and linearity

**Fig. 4:**
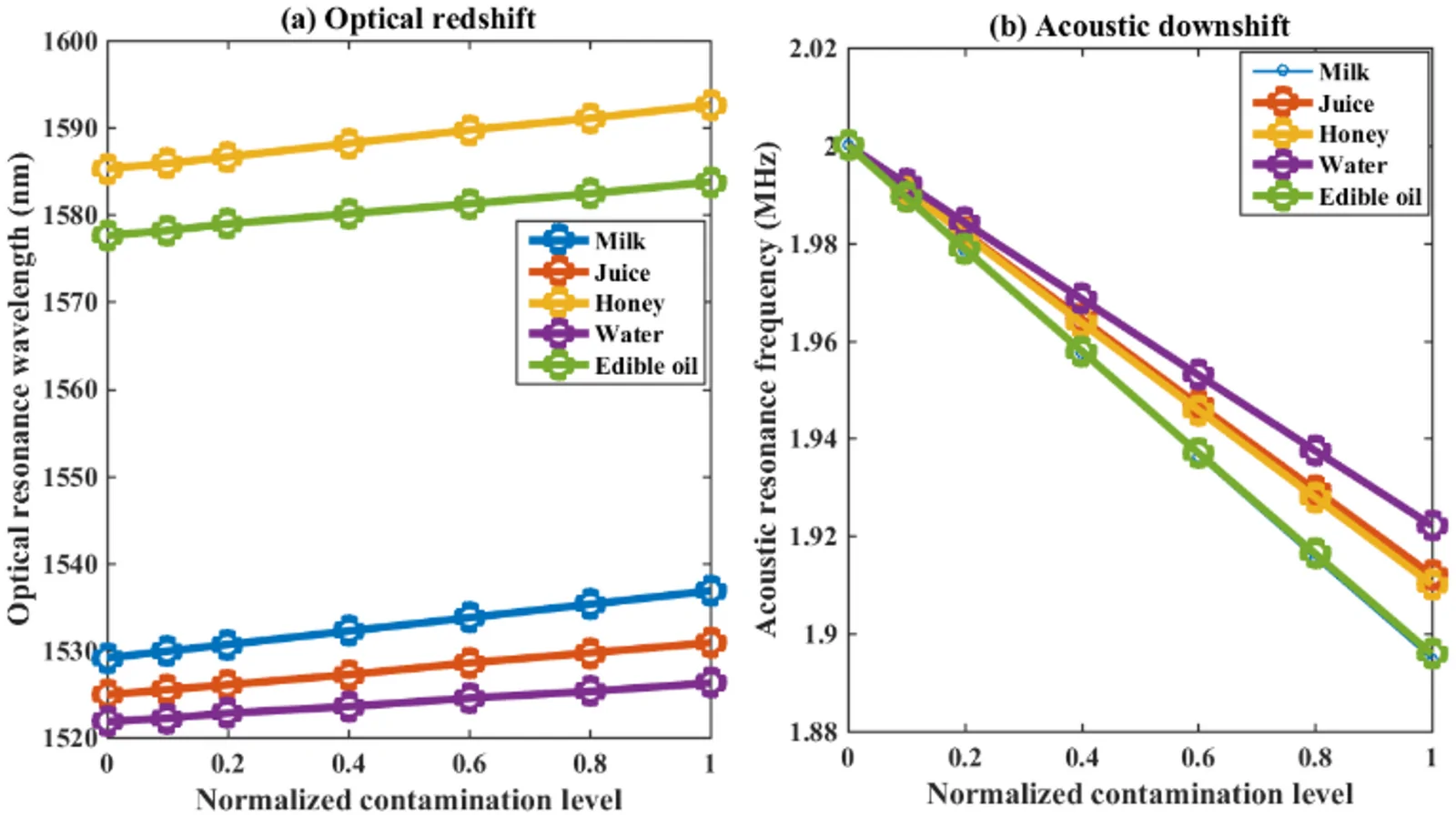
Dual-channel resonance evolution with normalized contamination level. The optical branch shows a monotonic redshift, while the acoustic branch shows a monotonic downshift, confirming complementary sensing trends within the same defect-based platform.

Figure 4 summarizes the resonance-position evolution with contamination level. The optical resonance increases monotonically with contamination, while the acoustic resonance decreases monotonically. The two trends have opposite signs, but both are systematic and strongly correlated with the normalized contamination level. This behavior confirms that the proposed sensor can track contamination-induced perturbations using two independent physical quantities.

The optical sensitivity ranged from 4.4025 to 7.6953 nm/fraction. Milk produced the strongest optical response, with a sensitivity of 7.6953 nm/fraction. This indicates that, under the present effective perturbation model, milk creates the largest optical path-length change in the defect cavity. Honey and edible oil also show strong optical responses, whereas water gives the lowest optical sensitivity.

The acoustic sensitivity magnitude ranged from 77.901 to 105.314 kHz/fraction. Milk again produced the largest response, with an acoustic sensitivity magnitude of 105.314 kHz/fraction. The negative acoustic slope should not be interpreted as a weaker response; it simply reflects the direction of the shift toward lower frequency. For performance comparison, the magnitude of the acoustic sensitivity is the relevant quantity.

The linearity of the response is one of the strongest features of the results. The optical coefficient of determination lies between 0.9989 and 1.0000, while the acoustic response reaches 1.0000 in the studied range. This means that the resonance shifts can be calibrated using simple linear relations, as defined in the Mathematical Formulation section, without requiring a complex nonlinear fitting model over the investigated contamination interval.

### Q-factor degradation as a secondary sensing indicator

**Fig. 5:**
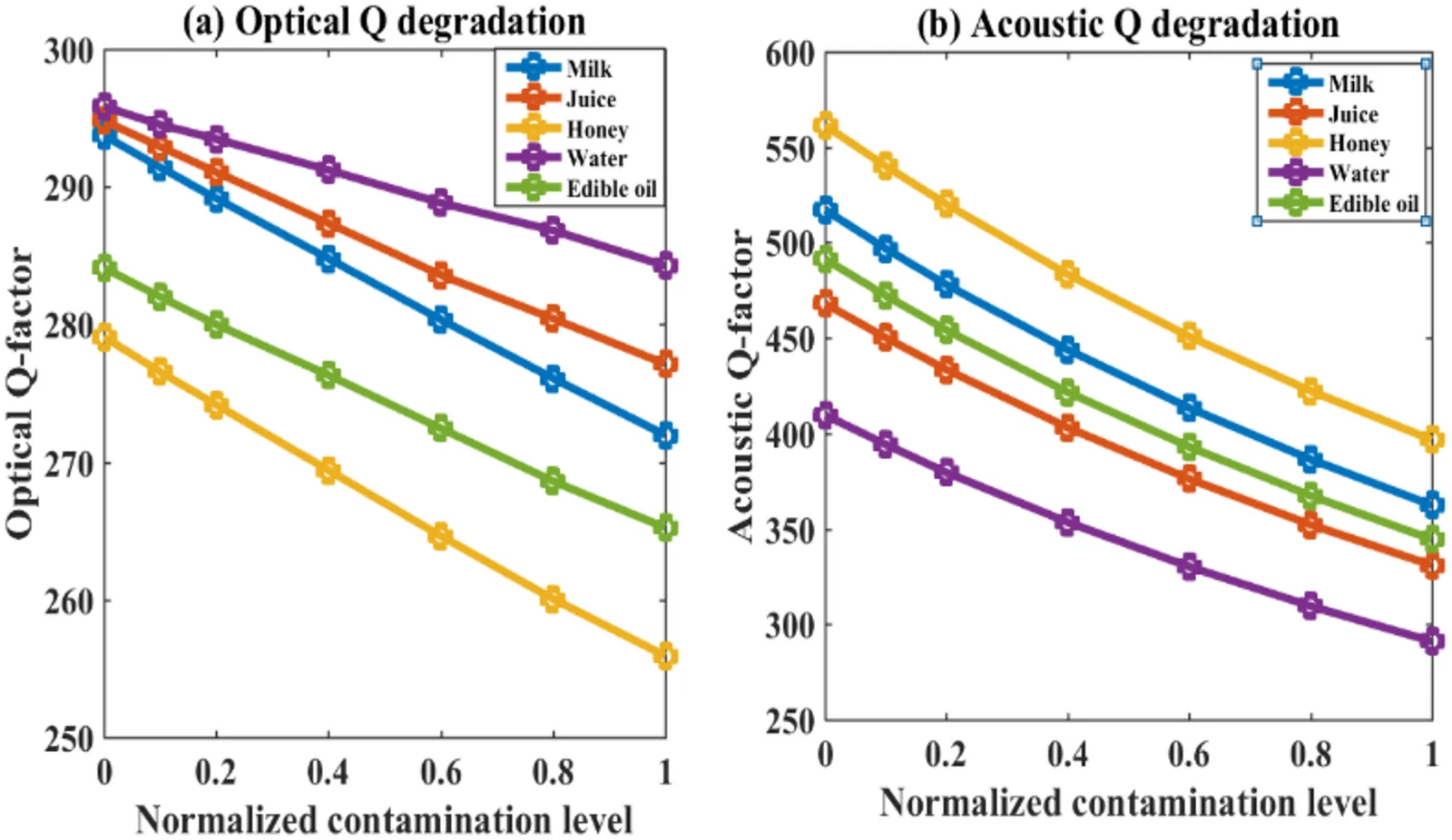
Optical and acoustic Q-factor degradation with increasing contamination level. The reduction in Q-factor indicates resonance broadening caused by increased optical loss and acoustic linewidth broadening in the contaminated defect region.

Figure 5 shows that both the optical and acoustic Q-factors decrease as the contamination level increases. This is a critical result because it shows that the sensing mechanism is not limited to resonance-position tracking. Contamination also modifies the resonance linewidth and the degree of energy confinement.

In the optical channel, Q-factor degradation is mainly associated with the increase in effective optical loss inside the defect layer. A larger loss term broadens the resonance and reduces the sharpness of the transmission peak. In the acoustic channel, the linewidth broadening follows the effective increase in acoustic damping represented in the model. In both cases, the reduction in Q-factor indicates that the defect mode becomes less sharply confined as the defect medium becomes more perturbed.

This additional information improves the physical interpretation of the sensing response. A shift-only sensor can report that the resonance moved, but it does not describe whether the mode quality was preserved or degraded. Here, the simultaneous shift and Q-factor reduction show that contamination changes both the phase condition and the loss/broadening behavior of the defect mode. This makes the response more informative and more suitable for multiparameter sensing analysis.

### Dual-channel sensitivity comparison and detection limit

**Fig. 6:**
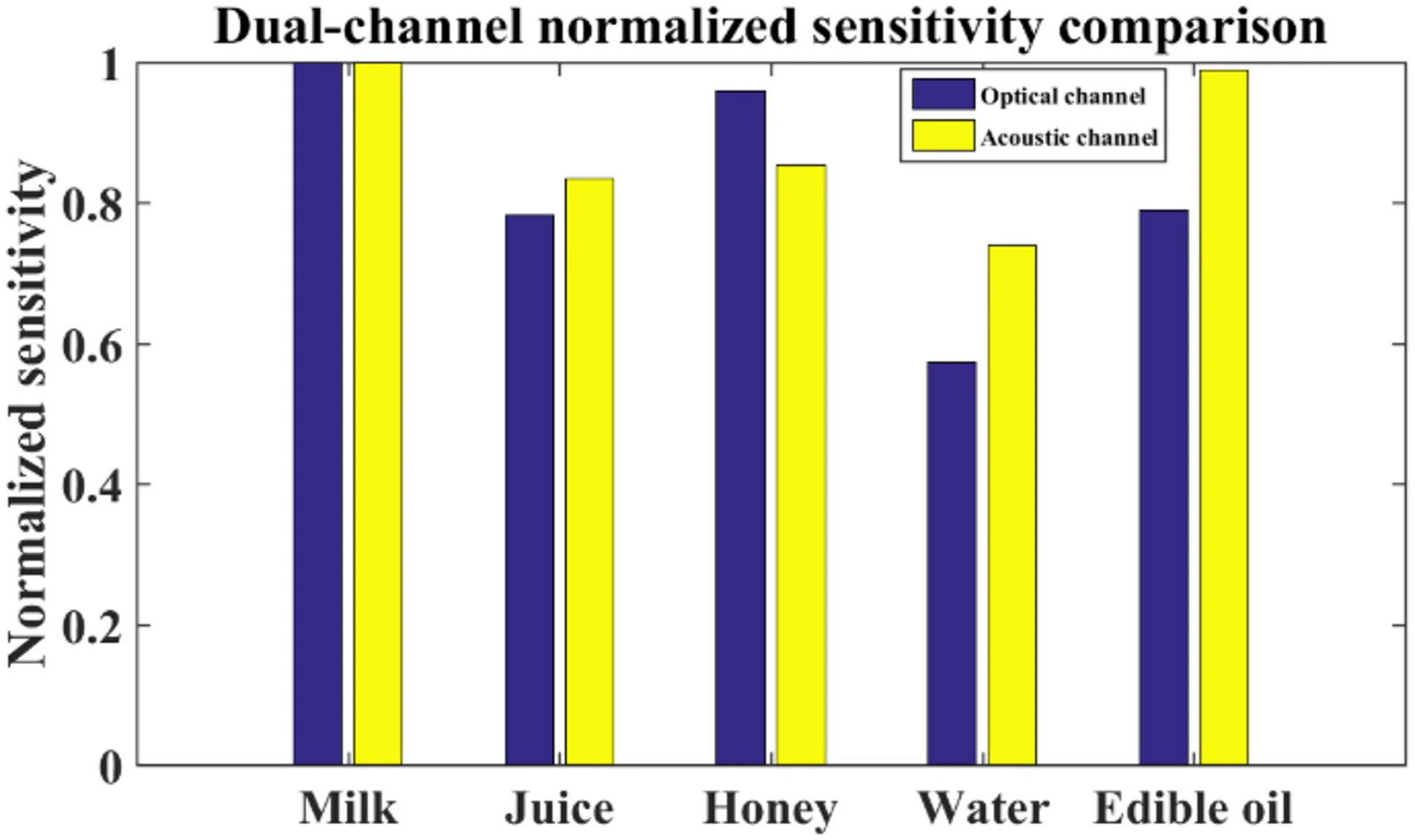
Normalized optical and acoustic sensitivity comparison for different food samples, highlighting the relative dual-channel response strength of the proposed phoxonic sensor.

Figure 6 compares the normalized sensitivities of the optical and acoustic channels. Normalization is necessary because the optical response is expressed in nm/fraction, while the acoustic response is expressed in kHz/fraction. The comparison shows that the two channels do not always rank the food samples in exactly the same way, which supports the usefulness of using both readouts rather than relying on a single spectral shift.

The estimated optical limits of detection range from 0.00390 to 0.03716 fraction, while the acoustic limits of detection range from 0.01558 to 0.01991 fraction. These values were obtained after introducing realistic noise floors, which prevents the ideal numerical fit from producing unrealistically zero detection limits. This is important for publication because it makes the detection-limit estimation more defensible. The LOD (Limit of Detection) values were calculated using assumed instrumental noise floors of 0.01 nm for the optical readout and 0.0005 MHz for the acoustic readout, together with the fitting residual uncertainty.

The optical branch provides the lowest estimated LOD in the best case, whereas the acoustic branch gives a narrower and more uniform LOD range across the investigated samples. This suggests that the optical response may be more sensitive for early perturbation tracking, while the acoustic response can provide a stable independent confirmation. The strength of the proposed sensor therefore lies in combining the two channels rather than declaring one of them universally superior.

### Optical–acoustic fingerprinting and dual-channel discrimination

**Fig. 7:**
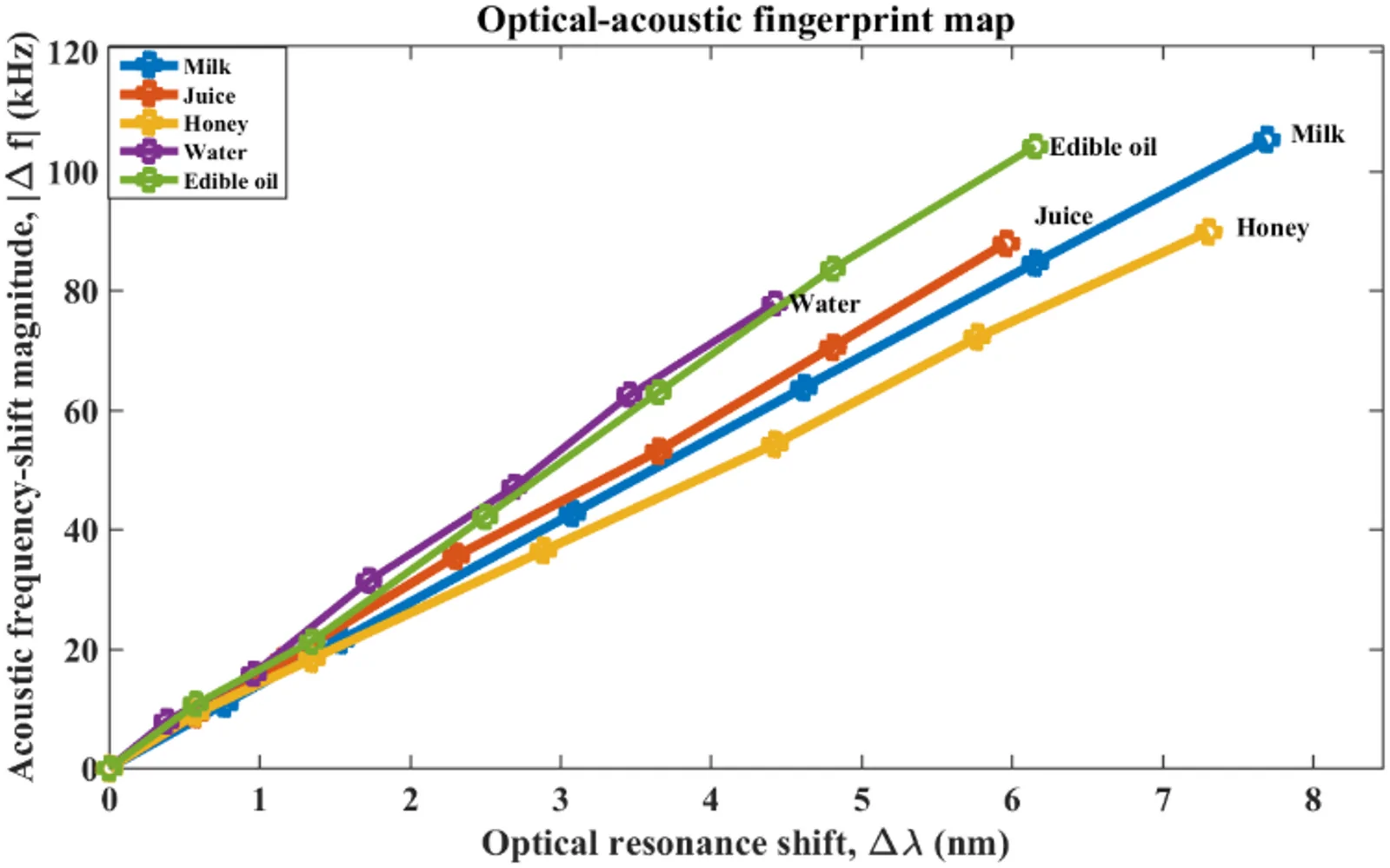
Dual-channel optical–acoustic fingerprint map of the proposed phoxonic food-contamination sensor. The optical resonance shift and acoustic frequency-shift magnitude form distinct trajectories for different food media, confirming the complementary discrimination capability of the dual-channel platform.

Figure 7 presents the optical–acoustic fingerprint map of the proposed defect-mode phoxonic sensor. In this representation, the optical resonance shift is plotted against the acoustic frequency-shift magnitude for each food sample as the normalized contamination level increases. The separated trajectories indicate that the optical and acoustic channels do not provide identical information. Instead, each food medium follows a distinct two-dimensional response path, confirming that the proposed platform can act as a dual-channel fingerprinting sensor rather than a conventional shift-only optical sensor. This result strengthens the discrimination capability of the model because the combined optical–acoustic response provides a richer physical description of the contamination-induced perturbation.

**Fig. 8:**
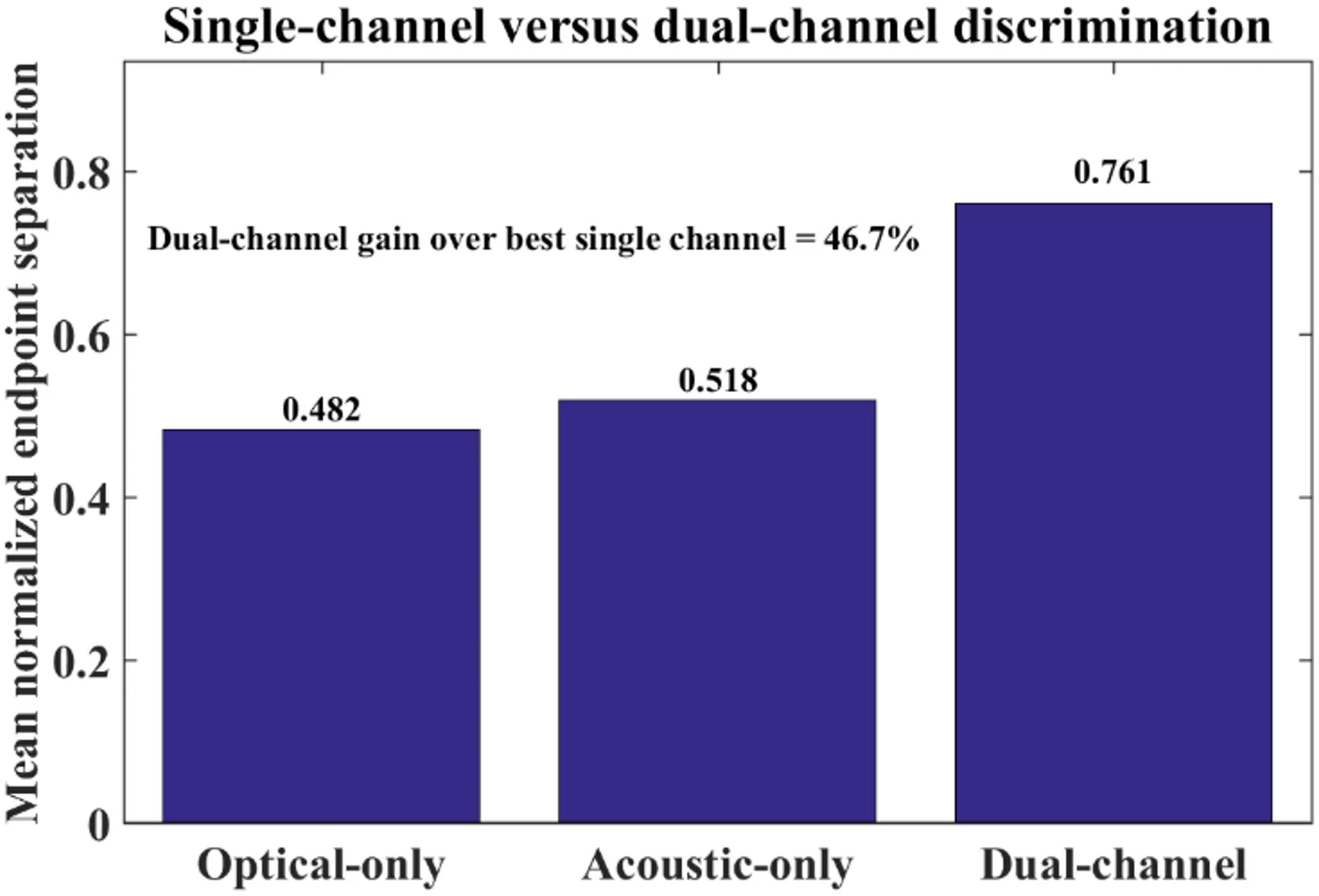
Single-channel versus dual-channel discrimination performance. The dual-channel optical–acoustic response provides higher mean normalized endpoint separation than either optical-only or acoustic-only sensing, with a 46.7% gain over the best single-channel result.

Figure 8 compares the discrimination performance of single-channel and dual-channel sensing. The optical-only and acoustic-only endpoint separations are 0.482 and 0.518, respectively, whereas the combined optical–acoustic response increases the separation to 0.761. This corresponds to a 46.7% gain over the best single-channel result. This improvement demonstrates the added value of the proposed phoxonic approach: the acoustic channel is not merely a duplicate of the optical response, but an independent complementary signature that enhances discrimination in the fingerprint space. Accordingly, the dual-channel configuration provides a stronger basis for contamination-sensitive detection than either optical or acoustic tracking alone.

### Phoxonic contamination index analysis

**Fig. 9:**
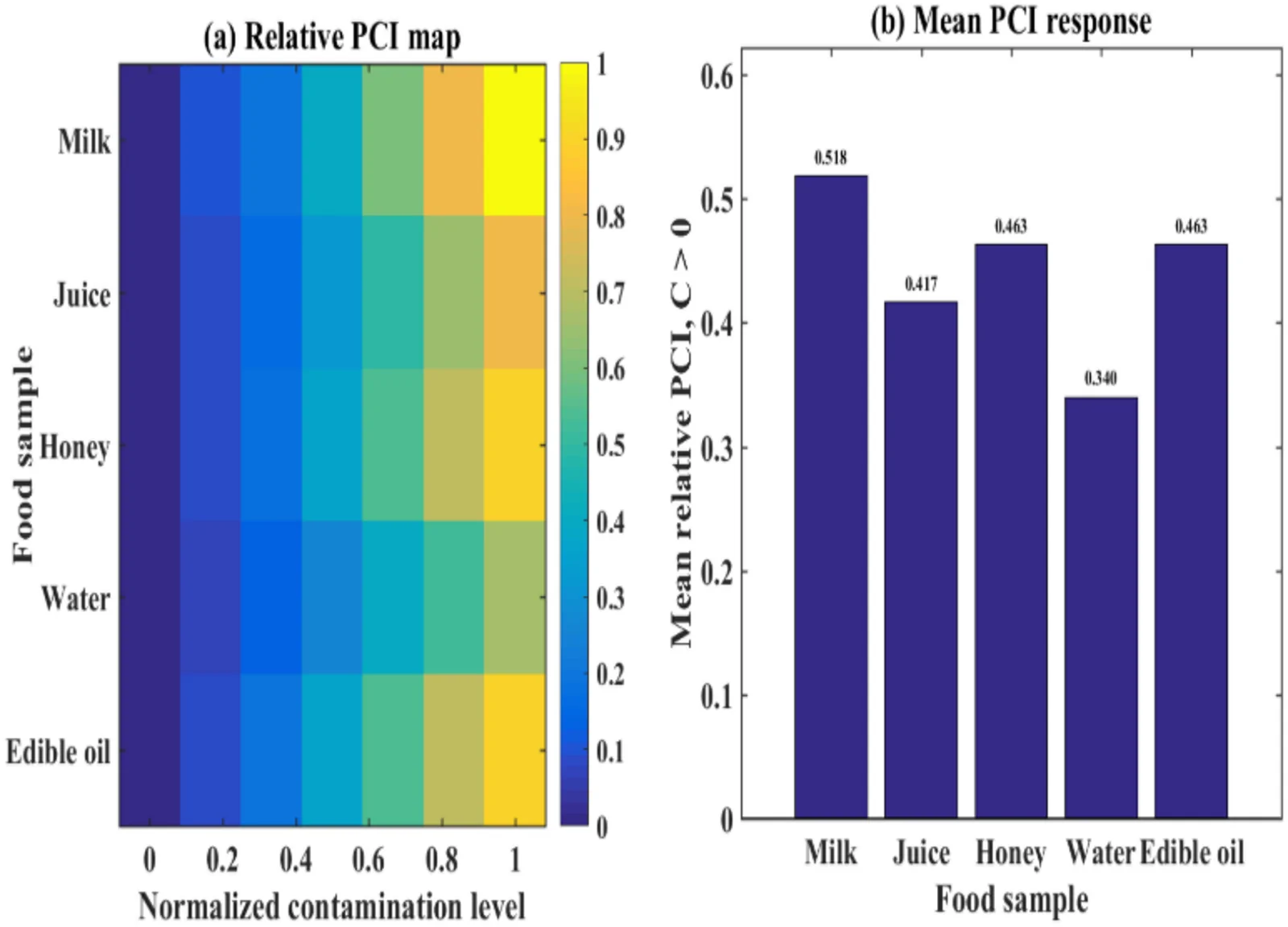
Relative phoxonic contamination index map and mean PCI response of different food samples. The PCI combines normalized optical and acoustic responses into a unified contamination-sensitive indicator and highlights the relative response strength of each food medium.

Figure 9 introduces the phoxonic contamination index (PCI), the relative PCI combines the normalized optical and acoustic responses into a single dimensionless indicator. Higher PCI values represent a stronger combined response to the imposed defect-medium perturbation.

The mean PCI response further reveals differences among the investigated food samples, with milk showing the strongest average response, followed by honey and edible oil, juice, and water. Therefore, PCI provides a useful additional metric for comparing the relative contamination sensitivity of different food media within the same phoxonic sensing framework. The phoxonic Contamination Index (PCI) was introduced to quantify the combined dual-channel discrimination between different samples. It was calculated by combining the normalized optical and acoustic response contrasts between two food samples. The optical contrast was obtained from the difference in resonance-wavelength shift, whereas the acoustic contrast was obtained from the difference in resonance-frequency shift. A higher PCI value indicates stronger dual-channel separability.

### Field localization and the active sensing region

**Fig. 10:**
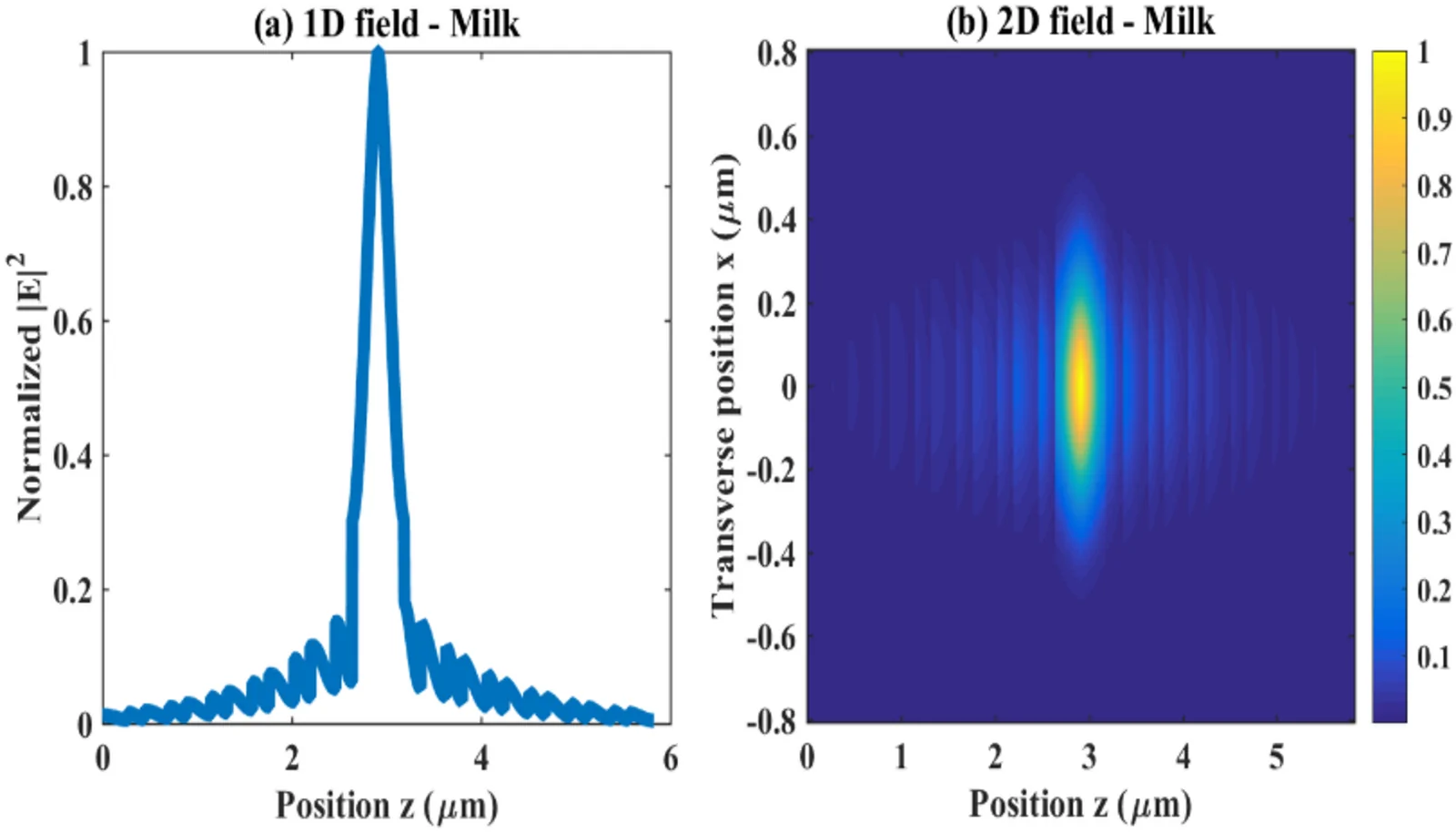
Representative one- and two-dimensional electric-field localization profiles around the central defect cavity. These profiles illustrate qualitative field confinement and are not full-wave FEM or FDTD simulations.

Figure 10 shows representative one- and two-dimensional electric-field profiles localized around the central defect cavity. This confinement confirms that the food-sensitive defect layer acts as the active optical sensing region, where changes in its effective refractive index and loss produce measurable resonance shifts and linewidth variations.

These field maps should be interpreted as representative confinement profiles rather than full electromagnetic simulations. They are included to clarify the physical role of the defect cavity and to support the transfer-matrix results. Full FEM (Finite Element Method) or FDTD (Finite-Difference Time-Domain) simulations would be required for exact spatial-field validation in a fabricated geometry.

#### Thickness tolerance and fabrication uncertainty

**Fig. 11:**
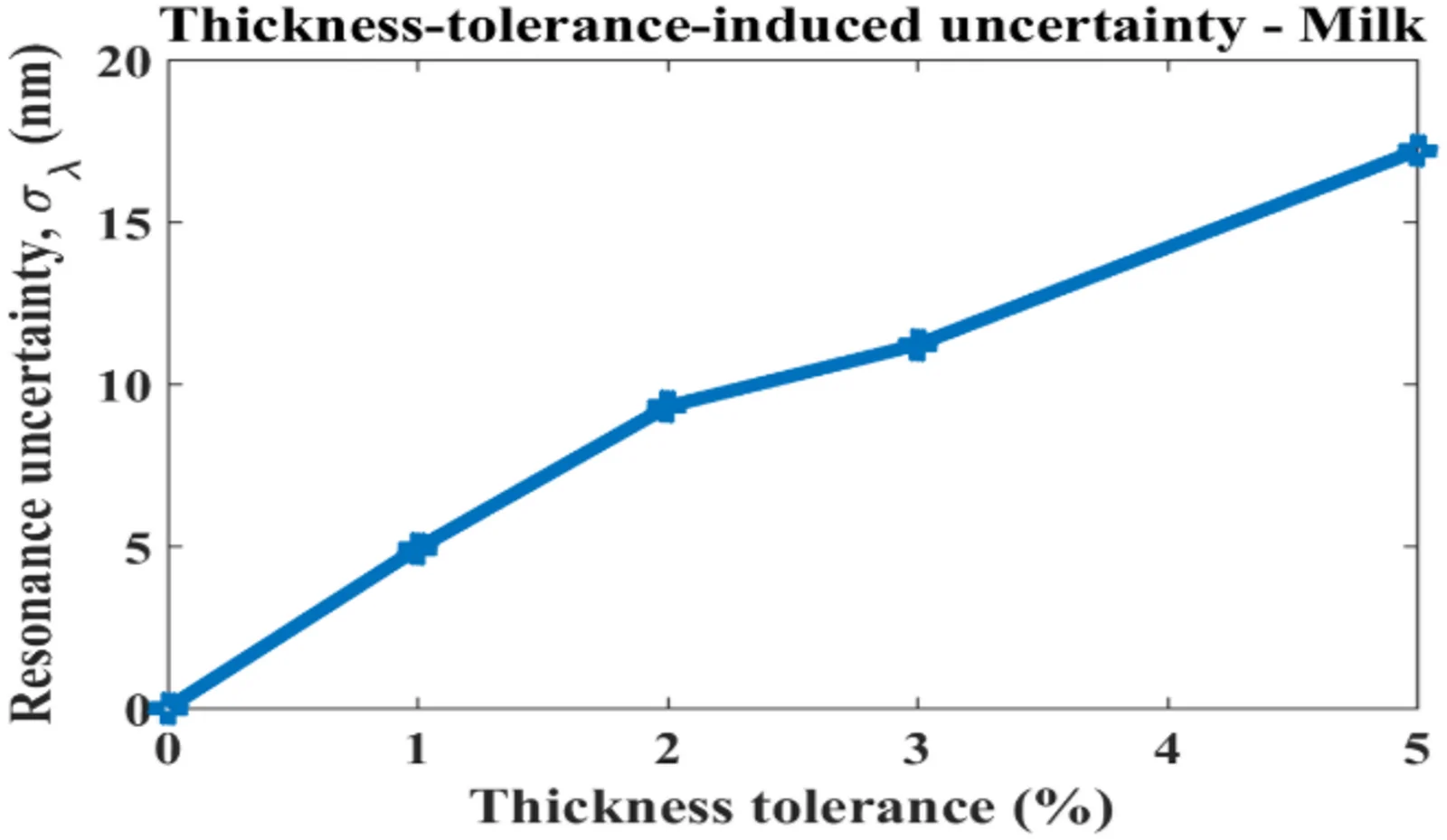
Thickness-tolerance-induced resonance uncertainty obtained using Monte Carlo perturbation of layer thicknesses. The increasing uncertainty reflects the sensitivity of the defect-mode position to fabrication deviations.

Figure 11 evaluates the effect of layer-thickness tolerance on the optical resonance position. The resonance uncertainty increases as the tolerance percentage increases, which is physically expected. The defect mode depends on the accumulated optical phase through the multilayer stack, and random deviations in layer thickness directly perturb this phase condition.

The tolerance analysis should not be described as proof of perfect fabrication robustness. Instead, it quantifies how much resonance uncertainty may be introduced by thickness deviations. This is a more realistic and publishable interpretation because practical multilayer sensors always experience some fabrication error. The key point is that the resonance remains trackable, but calibration and fabrication control become increasingly important as the tolerance level increases.

#### Surface roughness and linewidth broadening

**Fig. 12:**
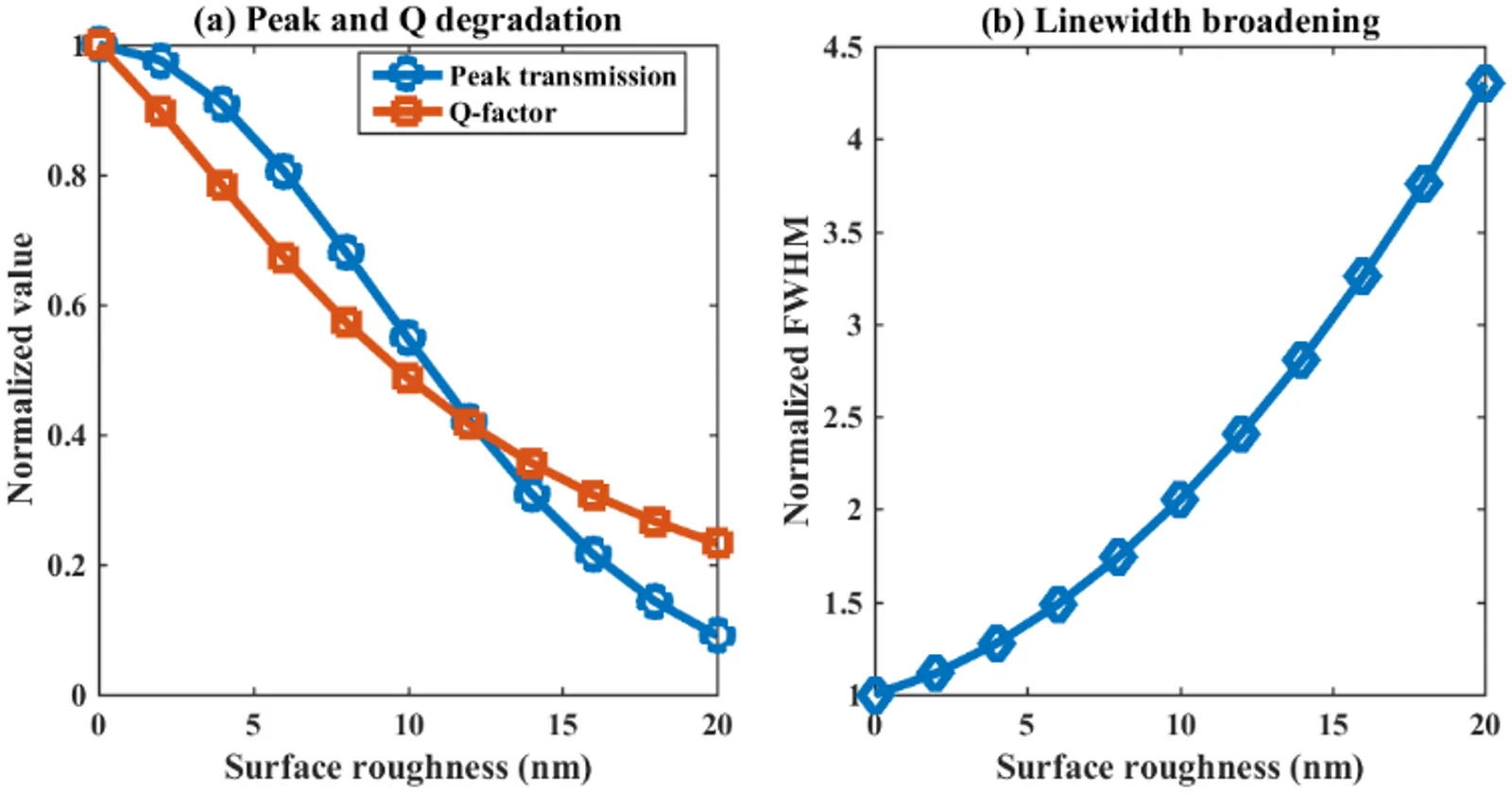
Influence of surface roughness on the normalized peak transmission, Q-factor, and resonance linewidth.

Figure 12 shows that increasing surface roughness reduces the normalized peak transmission and Q-factor while broadening the resonance linewidth. These trends are consistent with increased interface-scattering loss, which weakens coherent confinement and may reduce resonance contrast and peak-extraction precision.

#### Phase response and group-delay interpretation

**Fig. 13:**
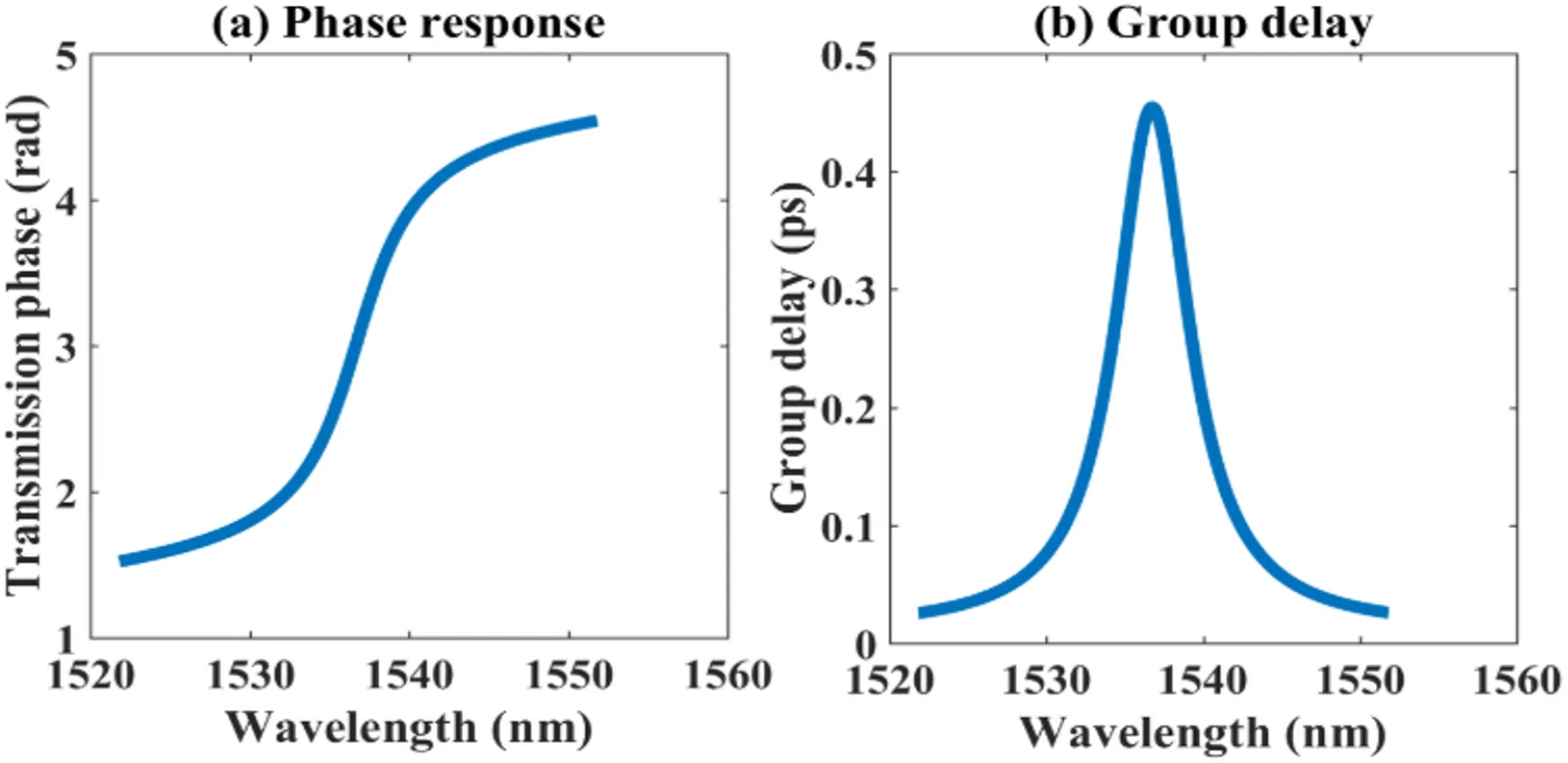
Transmission-phase response and group delay near the optical defect-mode resonance

Figure 13 shows a rapid phase variation and a localized group-delay maximum near the optical defect resonance. The maximum group delay reaches 0.4544 ps for the representative contaminated state, supporting the presence of a localized dispersive mode and an increased effective light–matter interaction time within the defect cavity.

#### Overall scientific interpretation

The results demonstrate a compact dual-channel phoxonic sensing concept in which the optical and acoustic branches provide complementary information. The optical channel converts effective refractive-index and loss changes into redshift and Q-factor degradation. The acoustic channel converts density and sound-velocity changes into frequency downshift and acoustic Q-factor degradation. These responses are physically connected through the same defect perturbation but are not redundant because they probe different material properties.

The strongest overall response was obtained for milk, with an optical sensitivity of 7.6953 nm/fraction and an acoustic sensitivity magnitude of 105.314 kHz/fraction. Across all samples, the high linearity and measurable detection limits indicate that the model provides a stable numerical framework for contamination-sensitive sensing analysis. Together, these analyses provide complementary physical and robustness indicators beyond resonance-shift tracking alone.

#### Model scope and limitations

The present work should be interpreted as a theoretical effective-medium study. The food-sensitive defect layer is described using effective optical and acoustic perturbation parameters, rather than direct biochemical reaction modeling, microbiological growth kinetics, or experimental contamination calibration. Since real food matrices are complex, similar optical or acoustic changes may arise from contamination, adulteration, natural compositional variation, turbidity, temperature changes, or storage-related degradation. Therefore, the proposed dual-channel response should be considered a contamination-sensitive physical fingerprint, not a direct identification of a specific biochemical or microbiological source. The results demonstrate the feasibility and physical consistency of the defect-based phoxonic sensing concept, while future work should validate the predicted trends using real food samples, controlled contamination protocols, and reference analytical methods. The reported LOD values depend on the assumed readout noise floor and should therefore be interpreted as theoretical estimates under the stated optical and acoustic resolution limits.

The normalized perturbation parameters serve as effective modeling variables for demonstrating the proposed sensing concept and are not designed to replicate the precise physicochemical characteristics of particular contaminated food samples. Establishing direct quantitative relationships will require future experimental calibration. Likewise, detailed acoustic finite-element analyses of field distributions, resonance frequencies, and modal behavior fall outside the scope of the present effective numerical proof-of-concept and will be addressed in subsequent validation studies. The normalized contamination parameter (C) is therefore defined as an effective phenomenological variable describing the combined contamination-induced changes in the optical and acoustic properties of the food-sensitive defect layer, rather than representing any single contamination mechanism. Quantitative benchmarking against established classification techniques, such as principal component analysis, cluster analysis, and machine-learning-based methods, is also beyond the present scope and will be considered following experimental validation.

The main sensing-performance parameters obtained from the proposed dual-channel phoxonic sensor, including optical sensitivity, optical LOD, mean optical Q-factor, acoustic sensitivity, and acoustic LOD for the investigated food samples, are summarized in Table 1.

**Table 1:** Summary of sensing performance.

Food sample	Optical sensitivity (nm/fraction)	Optical LOD (fraction)	Mean optical Q	Acoustic sensitivity (kHz/fraction)	Acoustic LOD (fraction)
Milk	7.6953	0.00390	283.93	105.314	0.01564
Juice	6.0177	0.02347	286.76	87.947	0.01783
Honey	7.3834	0.02618	268.55	89.897	0.01747
Water	4.4025	0.03716	290.71	77.901	0.01991
Edible oil	6.0800	0.03287	275.54	104.159	0.01558

The LOD values were estimated using optical and acoustic noise-floor values of 0.01 nm and 0.0005 MHz, respectively.

## Conclusion

This study presents a compact one-dimensional defect-mode phoxonic sensor for dual optical–acoustic characterization of food-contamination-induced property changes. The proposed design converts variations in the effective properties of the liquid food samples, which constitute the sensing medium inside the defect cavity, into two complementary responses: an optical resonance redshift related mainly to refractive-index and optical-loss changes, and an acoustic frequency downshift associated with density and sound-velocity variations. This combined response provides a more informative description of the sensing event than a single optical readout.

The numerical results show that the structure can track contamination-dependent changes in different food media with clear linear trends, measurable sensitivity, and noise-floor-based detection limits. The optical–acoustic fingerprint map produced distinguishable trajectories for the investigated samples, supporting the ability of the dual-channel framework to improve sample discrimination. The relative phoxonic contamination index also provided a unified indicator of contamination-induced changes, while the dual-channel endpoint separation reached 0.761, giving a 46.7% improvement over the best single-channel response.

The observed degradation of both optical and acoustic Q-factors indicates that contamination affects not only the resonance position but also the sharpness and confinement quality of the defect modes. In addition, the field-confinement analysis confirms the central defect cavity as the active sensing region, whereas the thickness-tolerance and surface-roughness analyses quantify the expected degradation trends under representative structural variations.

The proposed platform provides a physically interpretable approach for dual-channel food-quality monitoring using a compact phoxonic structure. As the study is based on an effective numerical model, future work should focus on calibration with real food samples, validation under temperature, humidity, turbidity, and matrix-complexity effects, and extension toward specific biochemical or microbiological contamination markers. The current samples were selected as representative examples to demonstrate the proposed numerical proof-of-concept. Additional liquid foods will be investigated in future experimental studies.

## Data Availability

The data generated and analyzed during the current study are available from the corresponding author upon reasonable request.
